# Effect of Heat Treatment under Different Atmospheres on the Bonding Properties and Mechanism of Ceramiziable Heat-Resistant Adhesive

**DOI:** 10.3390/polym16040557

**Published:** 2024-02-18

**Authors:** Qingke Wang, Jiadong Tao, Huawei Shan, Tangyin Cui, Jie Ding, Jianghang Wang

**Affiliations:** 1 School of Materials Science and Engineering, Wuhan University of Technology, Wuhan 430070, China; wangqingke@whut.edu.cn (Q.W.);; 2 System Design Institute of Hubei Aerospace Technology Academy, Wuhan 430040, China; 3 Shandong Industrial Ceramics Research and Design Institute Co., Ltd., Zibo 255100, China

**Keywords:** molybdenum-phenolic resin, heat-resistant, adhesive, ceramization, different atmospheres

## Abstract

In this study, a heat-resistant adhesive was prepared using molybdenum-phenolic (Mo-PF) resin as the matrix and TiB_2_ particle as the ceramizable filler for bonding Al_2_O_3_ ceramic substrates. Firstly, Fourier transform infrared (FTIR) was used to characterize the chemical structure of the Mo-PF. Subsequently, thermo gravimetric analysis (TGA) and shear strength testing were employed to investigate the effects of heat treatment in different atmospheres on the thermal stability and residual bonding properties of the adhesive. To further explore the bonding mechanism of the adhesive after heat treatment in different atmospheres, scanning electron microscopy (SEM), compressive strength testing, and X-ray diffraction (XRD) were utilized to analyze the microstructure, mechanical strength, and composition evolution of the adhesive at different temperatures. The bonding strength of Al_2_O_3_ joints showed a trend of initially decreasing and then increasing after different temperature heat treatment in air, with the shear strength reaching a maximum value of 25.68 MPa after treatment at 1200 °C. And the bonding strength of Al_2_O_3_ joints decreased slowly with the increase of temperature in nitrogen. In air, the ceramicization reaction at a high temperature enabled the mechanical strength of the adhesive to rise despite the continuous pyrolysis of the resin. However, the TiB_2_ filler in nitrogen did not react, and the properties of the adhesive showed a decreasing tendency with the pyrolysis of the resin.

## 1. Introduction

Engineering ceramic materials have been widely applied in fields such as automotive, aerospace, and nuclear power generation due to their outstanding resistance to oxidation, corrosion, excellent mechanical properties, and high temperature stability [1,2,3]. However, the high rigidity and brittle characteristics of engineering ceramic materials often pose challenges in the machining of complex components and the manufacturing of large-sized structures. Additionally, issues such as prolonged manufacturing time and high costs further limit the development of their engineering applications [4,5]. To overcome these challenges, finding effective joining methods has become key to the application of engineering ceramic materials [6,7,8]. Currently, common ceramic joining techniques include mechanical bonding [9], solid-state diffusion joining [10,11], glass or glass ceramic joining [12], and brazing [13]. However, these methods are associated with some potential structural drawbacks, such as stress concentration, increased structural weight, and issues related to mismatched thermal expansion coefficients. In comparison to traditional approaches, adhesive bonding technology has garnered attention due to its significant advantages [14]. The use of high-temperature adhesive for joining not only requires no special equipment and is easy to operate, but also can effectively alleviate the problem of stress concentration and maintain the integrity of the ceramic matrix. This bonding technique not only reduces manufacturing costs and enhances production efficiency but also fulfills the connection requirements for complex structures and large-sized ceramic products, offering a reliable and practical solution for the application of engineering ceramic materials. Therefore, the application prospect of adhesive bonding technology in the field of engineering ceramics is highly anticipated, which provides a strong support for the innovation and development of engineering ceramic materials.

Phenolic resin is a type of polymer compound formed by the condensation of phenolic compounds and aldehydes through acid or alkali catalysis [15,16]. Under inert atmospheres, it exhibits excellent resistance to combustion and high-temperature carbon residue, while also demonstrating good adhesive properties. Phenolic resin is commonly used as the matrix resin for heat-resistant adhesives, and exhibits outstanding mechanical properties, high-temperature resistance, and chemical stability. They can provide stable bonding performance in complex environments. Therefore, phenolic adhesives can meet various requirements in the field of engineering ceramic connections [17,18,19,20]. However, when the temperature surpasses 200 °C, the phenolic hydroxyl groups and methylene groups in the pure phenolic resin structure are prone to oxidation [21], impacting its thermal stability and bonding performance at high temperatures [22,23]. This limitation hinders its application in high-temperature fields. Therefore, the heat-resistant modification of phenolic resins and their prepared adhesives is particularly important.

The heat-resistant modification methods for phenolic resin primarily include chemical modification and physical modification. Chemical modification involves introducing new heat-resistant structures into the main chain of the phenolic resin during its preparation, for example, introducing elements like Mo, Si, B, Ti, and Zr to form covalent bond structures [24,25,26,27] and incorporating heat-resistant functional groups [28,29]. Introduction of molybdenum elements into the phenolic resin molecular chain is achieved by adding molybdic acid, ammonium molybdate, or organic molybdenum during the reaction process. Since conventional phenolic resins are connected by C-C bonds on the benzene ring, while molybdenum-modified phenolic resins are connected by stronger O-Mo-O bonds on the benzene ring, the thermal decomposition temperature and heat resistance of molybdenum-modified phenolic resins are improved compared to conventional phenolic resins [27]. Lin et al. [30] employed ammonium molybdate as the molybdenum modifier and synthesized thermosetting molybdenum phenolic resin through a two-step process. The weight residual rate of this resin at 800 °C was 71.3%, showing a 16% improvement in comparison to conventional phenolic resins. Li Shan et al. [31] synthesized a novel silane coupling agent, and then reacted it with phenol and formaldehyde to obtain a silicon-containing phenolic resin. It was shown that the oxidation resistance of the modified resin was greatly enhanced, and the residual content after oxidation at 1000 °C was much higher than that of the pure phenolic resin. Fengyi Wang et al. [32] prepared boron-modified phenolic resin using boric acid as a modifier, and the results indicated an improvement in the thermal stability of the boron-modified phenolic resin, with a residual charcoal rate of 69%. Zhang et al. [33] synthesized a novel titanium-modified phenolic resin using tetraisopropyl titanate, phenol, and formaldehyde. Compared to the unmodified phenolic resin, the maximum decomposition rate temperature increased by 43 °C, and the residual char yield at 850 °C increased by 11%. The physical modification of phenolic resin is primarily achieved by introducing inorganic fillers. These fillers can undergo redox reactions with the volatile components generated during the high-temperature pyrolysis of phenolic resin. This process enhances the pyrolysis temperature and residual charcoal rate of phenolic adhesives, meeting the requirements for the application of phenolic resin in high-temperature environments. Haddadi et al. [34] employed nano-SiO_2_ and B_4_C for the modification of phenolic resin, preparing high-resistant adhesives with excellent bonding strength to graphite materials in the range of 200 to 1000 °C. Wang et al. [35] incorporated ZrSi_2_ powder and B_4_C powder into a boron-phenolic resin matrix for ceramic material bonding. The experimental results indicated that the addition of inorganic fillers enhanced the thermal stability and mechanical properties of the phenolic adhesive, and the shear strength of the adhesive reached 36.6 MPa after 1200 °C treatment.

In the previous studies of high-resistant phenolic adhesives, most of them explored the high-temperature resistance and bonding strength of the adhesives, but the bonding mechanism after heat treatment in different atmospheres is rarely reported. In order to enhance the heat resistance of phenolic adhesives at high temperatures and investigate their bonding mechanism after heat treatment in two different atmospheres (air and nitrogen), this paper introduced molybdenum element into phenolic resin to synthesize a modified phenolic resin, and added TiB_2_ as ceramizable filler to prepare an organic–inorganic composite modified phenolic adhesive for bonding Al_2_O_3_ ceramic substrates. The thermal stability, mechanical properties, microstructure and phase evolution of the adhesive were analyzed with the help of appropriate testing methods, and the effects of heat treatment in different atmospheres on the adhesive were investigated.

## 2. Materials and Methods

### 2.1. Materials

Phenol, 37% aqueous solution of formaldehyde, anhydrous ethanol, sodium hydroxide, and molybdic acid were purchased from Shanghai Sinopharm Chemical Reagent Co., Ltd. (Shanghai, China). TiB_2_ powders were supplied by Shanghai Aladdin Biochemical Technology Co., Ltd. (Shanghai, China) with 98% purity and 4–8 µm particle size. The Al_2_O_3_ ceramic substrates with the dimensions of 40 × 20 × 5 mm and 20 × 20 × 5 mm were provided by Shenzhen Beilong Electronic Materials Factory (Shenzhen, China). All raw materials were directly used without further purification.

### 2.2. Testing and Characterization

The chemical modification of phenolic resin was investigated by Fourier transform infrared spectroscopy, on KBr pellets from 400 cm^−1^ to 4000 cm^−1^ by a Nicolet Nexus IR Spectra (Madison, WI, USA). The thermal stabilities of Mo-PF and ceramizable adhesive were analyzed using a comprehensive thermal analyzer (TGA, NETZSCH STA449F3, Bavaria, Germany) with a constant heating rate of 10 °C/min in air and nitrogen. An electronic universal material testing machine (Instron Model 5967, Instron, Norwood, MA, USA) was used to test the shear strength of Al_2_O_3_ joints and the compressive strength of the adhesive after heat treatment, with a loading rate of 2 mm/min. Five samples were tested under the same conditions. The microstructure of the adhesive was observed by field emission scanning electron microscopy (FESEM, Zeiss Ultra Plus, Oberkochen, Germany). 

### 2.3. Synthesis of Molybdenum–Phenolic (Mo-PF) Resin 

**Figure 1:**
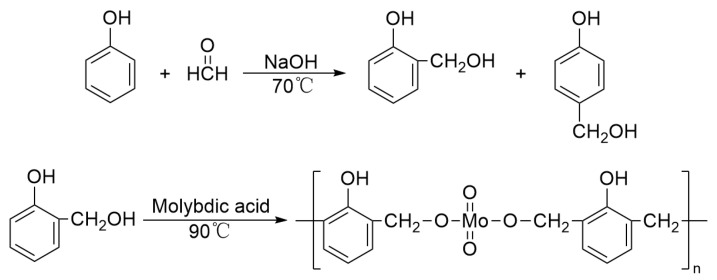
Schematic diagram of the synthesis process of the Mo-PF [36].

In accordance with the methods outlined in the referenced literature [36], Mo-PF and unmodified phenolic resin (PF) were synthesized. The study revealed that the reaction between molybdic acid and phenol was difficult, while the reaction with hydroxymethylphenol proved to be more facile. Therefore, the modification of phenolic resin with molybdic acid was conducted in two steps: firstly, the generation of hydroxymethylphenol from the reaction between phenol and formaldehyde under alkaline catalysis, followed by the reaction of molybdic acid with hydroxymethylphenol to synthesize Mo-PF (Figure 1). The preheated phenol, a 37% formaldehyde solution, and NaOH were added to a three-neck round-bottom flask equipped with a thermometer, reflux condenser, and mechanical stirrer in a molar ratio of 1:1.2:0.15. The mixture was stirred, and refluxed for 1 h at 70 °C. Subsequently, 0.06 mol of molybdic acid was added, and the temperature was raised to 90 °C for an additional 1 h reflux, during which the color changed from yellow to deep green. Finally, black Mo-PF can be obtained by removing water under vacuum at 100 °C. Unmodified PF was prepared using the same process without the addition of molybdic acid.

**Figure 2:**
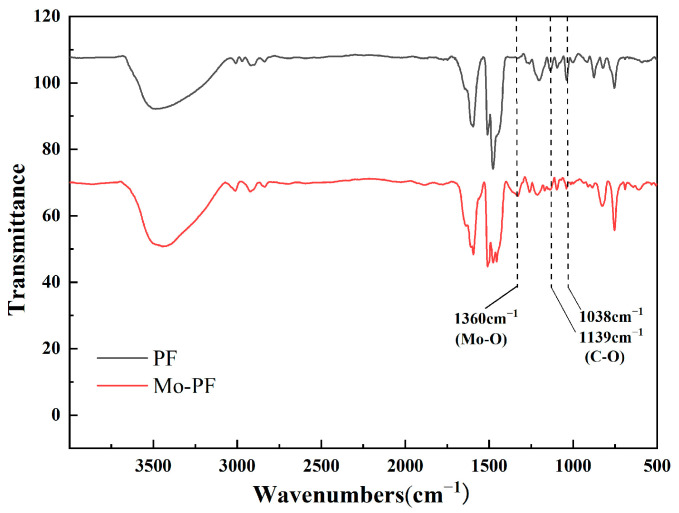
IR spectra before and after modification of phenolic resin.

The IR spectra of pure phenolic resin and modified phenolic resin are shown in Figure 2 to confirm the successful synthesis of Mo-PF. Compared with the IR spectrum of PF, the IR spectrum of Mo-PF has a characteristic absorption peaks at 1360 cm^−1^, corresponding to the asymmetric vibration of Mo-O [37,38]. The absorption peaks of aliphatic C-O stretching vibration are at 1139 cm^−1^ and 1038 cm^−1^. The characteristic peaks of modified phenolic resin in this region are significantly weakened, as the C-O-C bonds between the benzene rings within the pure phenolic resin are replaced by Mo-O-Mo, resulting in a reduction of ether bonds.

The infrared absorption peaks, except for those related to modified phenolic resin in this region, remain largely unchanged. The distribution of characteristic peaks is outlined as follows: the O-H peaks of the phenol group and -CH_2_OH exhibit a broad peak around 3500 cm^−1^; the C-H stretching vibration absorption peaks in the -CH_2_- region range from 2800 cm^−1^ to 2950 cm^−1^; the stretching vibration peaks of the C=C double bond in the benzene ring appear at 1610 cm^−1^ and 1510 cm^−1^; the distinctive absorption peaks of C-O in the phenolic group are identified at 1270 cm^−1^; and the ortho-substituted and para-substituted peaks of the benzene ring are observed at 756 cm^−1^ and 874 cm^−1^. All these absorption peaks are consistent with the synthesis results of phenolic resin reported elsewhere [39,40].

### 2.4. Preparation of the Adhesive and Al_2_O_3_ Joints

**Figure 3:**
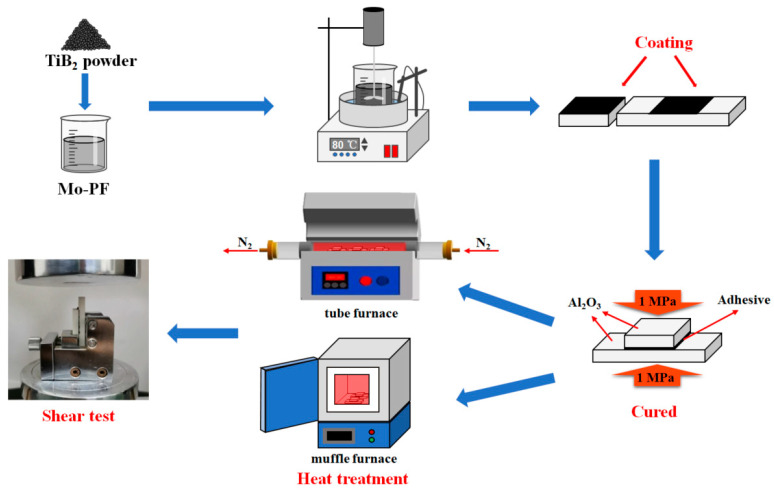
Preparation process of ceramizable adhesive and Al_2_O_3_ joints.

The preparation process of ceramizable adhesive and Al_2_O_3_ joints is shown in Figure 3. Firstly, the Mo-PF was preheated to 80 °C in a constant temperature water bath equipped with a mechanical stirring device. Subsequently, an equivalent mass of TiB_2_ particles was introduced, and the mixture was stirred for an additional 2 h at 80 °C to ensure uniform dispersion of TiB_2_ within the adhesive system. Before bonding, the Al_2_O_3_ ceramic substrates of two different sizes were ultrasonically cleaned in anhydrous ethanol for 0.5 h and then dried in the oven. Then, the adhesive was applied through the scraper onto the surface of the Al_2_O_3_ ceramic substrates to achieve follow-up firm bonding. Then, the obtained joints were cured under the pressure of 1 MPa at 120 °C for 1 h, 150 °C for 3 h, and 170 °C for 1 h. After the curing process, the Al_2_O_3_ joints were placed in a muffle furnace or tube furnace. They were heated to the required temperature at a rate of 10 °C/min, maintained at this temperature for 1 h, and subsequently cooled to room temperature within the furnace.

## 3. Results and Discussion

### 3.1. Thermo Gravimetric Analysis of Mo-PF and the Adhesive

**Figure 4:**
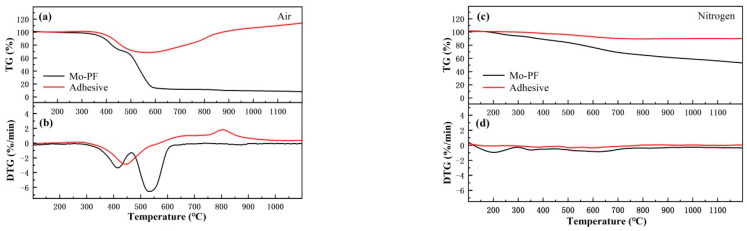
TG and DTG curves of Mo-PF and the adhesive under different conditions. (**a**) TG curves in air; (**b**) DTG curves in air; (**c**) TG curves in nitrogen; (**d**) DTG curves in nitrogen.

Adhesives with high weight retention often have good heat-resistant properties and high-temperature bonding strengths, which is conducive to their applications in high-temperature environments. Thermo gravimetric analysis (TGA) is used to explore the thermal stability of Mo-PF and the adhesive. One notable advantage lies in its ability to illustrate the correlation between weight loss percentage and temperature through the resulting curve. This enables a more intuitive evaluation of the material’s thermal stability [41]. Figure 4 is the TG/DTG curves of Mo-PF and the adhesive in air and nitrogen at a heating rate of 10 °C/min.

The TG and DTG curves in the air atmosphere are shown in Figure 4a,b. The weight of Mo-PF decreases with increasing temperature, while the weight of ceramizable adhesive exhibits an overall trend of initially decreasing and then increasing. In the temperature range of RT-320 °C, the adhesive exhibits relatively stable weight, while Mo-PF loses about 1.7% of its weight due to the volatilization of residual solvent and small-molecule gases in the resin matrix. From 320 °C to 550 °C, the weight of both samples decreases sharply, indicating that the resin has undergone intense pyrolysis in this temperature range. The molecular chain of phenolic resin is broken by heating and breaks into larger molecular segments. These macromolecular segments further undergo cleavage or oxidation reactions in the heated state, producing methane, CO, CO_2_, H_2_, H_2_O, and other small molecular gases and causing weight loss [42,43]. At this stage, the effect of filler on the heat resistance of the adhesive is not obvious, and the effect of temperature on the adhesive is mainly manifested in the destruction of the resin. When the temperature reaches about 550 °C, the pyrolysis rate of the resin reaches the maximum, and the ceramizable filler in the adhesive begins to react with the resin pyrolysis products to produce corresponding oxides, which makes up for the weight loss caused by the resin pyrolysis to a certain extent. At about 850 °C, the ceramization reaction of the filler continues, and the growth rate of the adhesive’s weight decreases slightly, which may be caused by the volatilization of the oxidation product of TiB_2_. Finally, the residual rates of Mo-PF and the adhesive at 1200 °C are 8.4% and 114.5%, respectively.

The TG and DTG curves in the nitrogen atmosphere are shown in Figure 4c,d. As the temperature increases, both Mo-PF and the adhesive exhibit a gradual overall decline in weight. In comparison, the weight loss of both in a nitrogen atmosphere is not as significant as that observed in an air atmosphere. In the temperature range of RT-300 °C, the weight of the adhesive is relatively stable, and the weight loss of Mo-PF is about 5.3%; this is due to the evaporation of residual water after curing in the resin and the further condensation and dehydration of the resin. After this temperature point, the fastest weight loss temperature of Mo-PF is about 620 °C, which is nearly 100 °C higher than the fastest weight loss temperature in the air, and the fastest weight loss temperature of the adhesive is about 610 °C, which is nearly 150 °C higher than the fastest weight loss temperature in the air. Moreover, the TG curve of the adhesive does not show a significant trend of weight increase after 700 °C, indicating that the inorganic particles mostly act as an inert filler and do not react as much as in the air, and only a small number of TiB_2_ particles may react with the resin pyrolysis products to maintain the weight of the adhesive. Finally, the weight residual rates of Mo-PF and the adhesive at 1200 °C are 53.8% and 90.1%, respectively. In summary, the thermal stability of Mo-PF in nitrogen surpasses that in air. On the other hand, the weight retention rate of the ceramicizable adhesive at a high temperature is higher in air compared to nitrogen.

### 3.2. Bonding Properties of Al_2_O_3_ Joints

**Figure 5:**
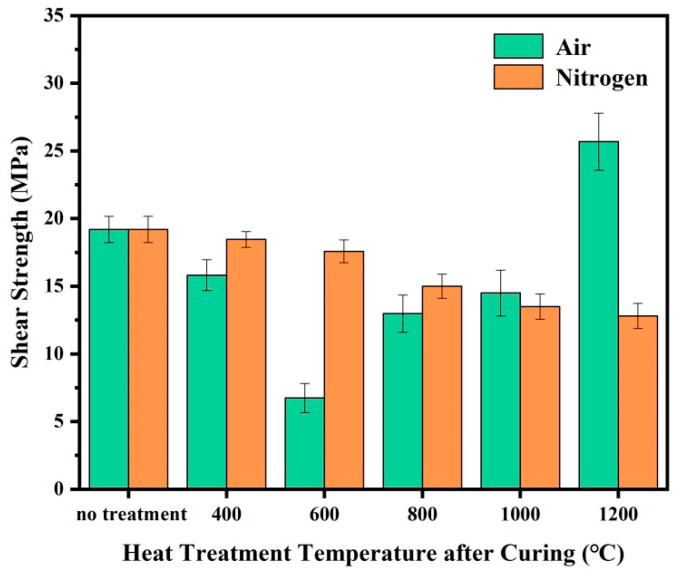
The shear strength of Al_2_O_3_ joints bonded by the adhesive after heat treatment at different temperatures and atmospheres.

The bonding properties of the joints were evaluated by testing the shear strength after treatment at different temperatures. Figure 5 shows the room temperature shear strength of the Al_2_O_3_ joints bonded with the prepared adhesive after heat treatment in different atmospheres of 400~1200 °C. The shear strength of the adhesive in air shows a decreasing trend with increasing temperature (RT-600 °C), followed by an increase (600–1200 °C). In nitrogen, the shear strength of the adhesive exhibits a gradual decrease with increasing temperature. The shear strength of the adhesive after curing is 19.20 MPa, while the shear strength decreases to 15.82 MPa and 18.52 MPa after treatment at 400 °C in air and nitrogen, respectively. At 600 °C, the shear strength of the adhesive in air decreases to a minimum of 6.74 MPa, while in nitrogen, it decreases to 17.58 MPa. The variation trend of shear strength for the adhesive is the same before 600 °C, indicating that the temperature’s influence on both is related to the pyrolysis of organic components. At 600 °C, however, the adhesive exhibits higher shear strength in nitrogen than in air. This is due to the lighter pyrolysis reaction of the phenolic resin matrix in nitrogen compared to air. Nitrogen provides a certain atmosphere protection for the adhesive at this temperature, while the presence of O_2_ intensifies the breaking of large molecular chains in the resin, leading to a significant decrease in mechanical properties. After being treated at 800 °C, the shear strength of the adhesive in air increases to 12.97 MPa, while the adhesive in nitrogen decreases to 15.01 MPa. As the temperature further increases, the shear strength of the adhesive treated in an air atmosphere continues to grow, reaching a maximum value of 25.68 MPa at 1200 °C. In contrast, the shear strength of the adhesive treated in a nitrogen atmosphere decreases to 12.80 MPa. This indicates that TiB_2_ particles can compensate for the mechanical deficiencies of the adhesive after high-temperature treatment in air, significantly improving the adhesive performance. Conversely, the enhancing effect of TiB_2_ particles on the adhesive strength is not pronounced in nitrogen, just as an inert filler role.

### 3.3. Fracture Morphology Analysis of Al_2_O_3_ Joints

**Figure 6:**
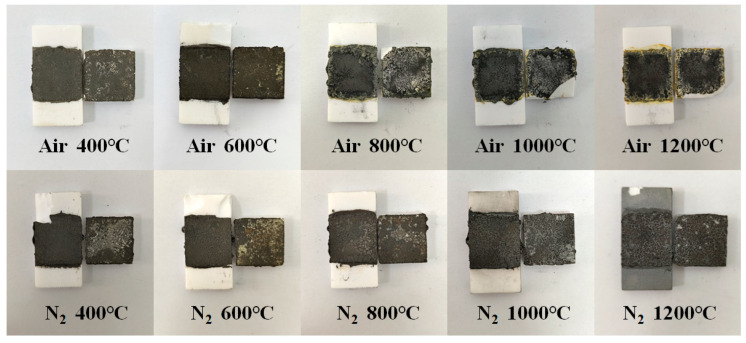
The physical photos of each Al_2_O_3_ joint after shear strength test.

The macroscopic morphology of the fracture was analyzed in the photos of the Al_2_O_3_ joints after the shear strength test in Figure 6. It can be observed that the failure models of ceramic joints treated under different conditions are distinct. After air treatment at 400 °C, the shear fracture surface exposed white ceramic, indicating a coexistence of interfacial and cohesive failure models. After air treatment at 600 °C, cohesive failure predominates, possibly due to numerous thermal defects within the adhesive that seriously impair the structural integrity of the bonding layer, resulting in a substantial reduction in matrix strength. As the temperature further rises to 800 °C, oxidation and melting of the inorganic filler effectively compensate for resin defects, significantly enhancing the strength of the adhesive. In addition, although some chemical bonds are formed at the interface between the adhesive and the ceramic, they are insufficient to match the strength of the adhesive, making interfacial failure the primary failure model. When the heat treatment temperature reaches 1000 °C and above, the shear fracture surface of the adhesive joint exhibits a mixed failure model, including interfacial failure, cohesive failure, and ceramic failure, indicating that the Al_2_O_3_ joint has achieved a higher bonding strength. This may be attributed to the reaction between B_2_O_3_ and Al_2_O_3_ at the interface between the adhesive and ceramic substrate, forming a gradient layer of 9Al_2_O_3_·2B_2_O_3_ [44]. As a result, the strength of the adhesive layer and the interface chemical bonding are both reinforced. Under nitrogen conditions, after treatment at 400 °C, the main failure model on the fracture surface of the adhesive joint is interfacial failure, indicating that the resin matrix’s strength remains significant. With increasing temperature, the failure model gradually shifts from a coexistence of interfacial and cohesive failure to cohesive failure predominance, with no ceramic failure. This suggests that TiB_2_ powder primarily serves as inert filler, does not undergo significant oxidation, and does not react with the ceramic substrate at the interface. The adhesive performance relies mainly on the mechanical strength of the phenolic resin matrix.

**Figure 7:**
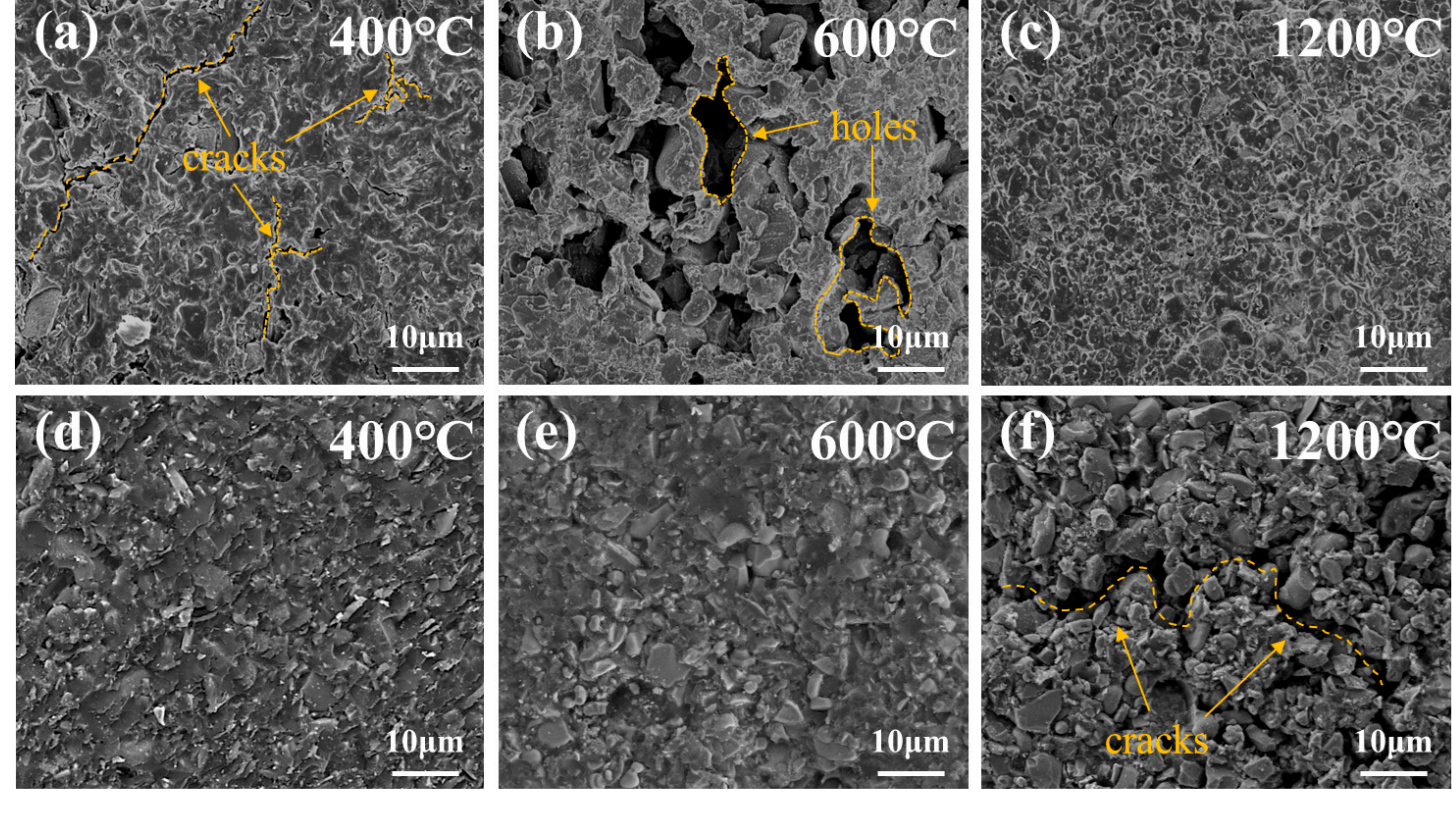
SEM fracture surface micrographs of the adhesive after heat treatment at different temperatures and atmospheres. (**a**) 400 °C in air; (**b**) 600 °C in air; (**c**) 1200 °C in air; (**d**) 400 °C in nitrogen; (**e**) 600 °C in nitrogen; (**f**) 1200 °C in nitrogen.

The fracture microstructure of the Al_2_O_3_ joints bonded by the adhesive after heat treatment under different temperatures and atmospheres are shown in Figure 7. Three temperature points—initial pyrolysis temperature in air (400 °C), temperature after intense pyrolysis (600 °C), and upper limit temperature on the TGA curve (1200 °C)—were selected as reference points for conducting microscopic analysis on the samples. After heat treatment at 400 °C in air, significant cracks and voids appeared on the adhesive surface due to the escape of small molecules and resin volume shrinkage. As the temperature reached 600 °C, resin pyrolysis intensified, and the small cracks gradually increased into larger holes. After heat treatment at 1200 °C, some TiB_2_ reacts with the pyrolysis products of the resin or gases in the air, forming TiO_2_ and B_2_O_3_ to effectively restrict volume shrinkage and crack formation in the bonding layer caused by the release of gas molecules. B_2_O_3_, in a liquid state at high temperature, exhibits excellent wetting properties and chemical compatibility with the carbon matrix. Once micro-cracks form at the bonding interface, a portion of the molten B_2_O_3_ migrates to the crack tip to prevent propagation [45,46,47]. This self-repairing effect effectively compensates for cracks and pores in the adhesive, resulting in an internally dense structure and consequently improving the mechanical properties of the adhesive. In contrast, after heat treatment at 400 °C in nitrogen, the fracture surface of the adhesive was very dense, and the TiB_2_ particles were tightly wrapped by the resin. The damage of the adhesive by shear force mainly focused on the resin matrix. The presence of TiB_2_ particles can withstand loads and inhibit crack formation, helping to prevent further crack propagation and positively contributing to maintaining bonding strength [48]. However, when the temperature reached 600 °C, some particles on the fracture surface began to appear, which was caused by the decrease in mechanical strength after the pyrolysis of phenolic resin and the weakening of the interface binding force between the particles and resin matrix. More seriously, after heat treatment at 1200 °C, a large number of TiB_2_ particles were observed on the surface, accompanied by the formation of large cracks, indicating further resin pyrolysis and a significant reduction in the overall strength of the adhesive.

### 3.4. Compression Strength of the Adhesive

Combined with the shear strength at each temperature and the macroscopic morphology of the fracture surfaces between the Al_2_O_3_ substrates, it can be seen that the bonding strength of the Al_2_O_3_ ceramic is closely related to the thermal and mechanical properties of the adhesive itself. In order to further explore the bonding mechanism of the phenolic adhesive, the curing blocks of Mo-PF and the adhesive were prepared, and the three temperature points of 400 °C, 600 °C, and 1200 °C were also selected as reference points for heat treatment in different atmospheres. The cured blocks of Mo-PF and adhesive were prepared and heat-treated in different atmospheres.

**Figure 8:**
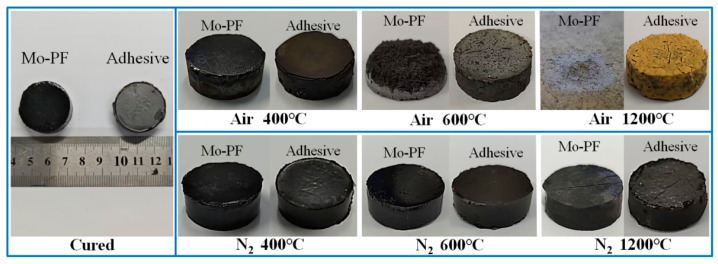
The physical photos of the cured blocks of Mo-PF and the adhesive before and after heat treatment under different conditions.

Figure 8 shows the physical photos of the cured blocks of Mo-PF and the adhesive before and after heat treatment at different temperatures and atmospheres. After air treatment at 400 °C, the surface of the cured Mo-PF resin block exhibited subtle cracks, while the adhesive block remained intact without apparent defects. At 600 °C, the cured Mo-PF resin block underwent extensive pyrolysis, losing nearly half of its weight and structural integrity, resulting in a powdery surface. At the same time, the adhesive block displayed a multitude of dense and conspicuous cracks on its surface. After treatment at 1200 °C, the Mo-PF resin block in air disappeared entirely, leaving a white powder residue, while the adhesive block surface turned yellow, showing cracks but maintaining structural integrity. Under nitrogen atmosphere conditions at 400 °C and 600 °C, both types of cured blocks displayed no significant surface alterations, maintaining their structural integrity. However, at 1200 °C in a nitrogen atmosphere, the Mo-PF resin block exhibited large cracks, and the adhesive block surface showed numerous small cracks. Although a small degree of pyrolysis occurred, there were no evident structural collapses.

The observed phenomena can be explained by considering the pyrolysis mechanism of phenolic resins. At 400 °C in air, the initial subtle cracks on the cured Mo-PF resin block may be attributed to the beginning of depolymerization and the release of volatile compounds. The intact surface of the adhesive block suggests that the incorporation of TiB_2_ filler enhances the overall stability of the material at this temperature. The significant weight loss and structural deterioration of the cured Mo-PF resin block at 600 °C in air indicate an intensified thermal degradation process. This can be associated with the cleavage of C-O and C-C bonds in the phenolic resin polymer network, leading to the formation of volatile species and the powdery residue observed on the surface. In contrast, the adhesive block exhibit dense and conspicuous cracks without structural collapse, suggesting that the addition of TiB_2_ filler may reduce stress concentration and enhance the thermal stability of phenolic resin to a certain extent. The complete disappearance of Mo-PF resin block and the color change of the adhesive block at 1200 °C in air indicate an advanced stage of pyrolysis. The observed yellowing of the adhesive block may be caused by thermal interaction or oxidation reaction between TiB_2_ filler and the surrounding matrix. The stability of the two cured blocks at 400 °C and 600 °C in a nitrogen atmosphere shows that the inert environment has a certain protective effect that can prevent or minimize the pyrolysis of Mo-PF. However, at 1200 °C, the appearance of cracks in the Mo-PF resin block and the development of small cracks in the adhesive block indicate that even in a nitrogen atmosphere, thermal stress and some degree of pyrolysis occur in the materials.

The mass variation and the compressive strength of Mo-PF and the adhesive cured blocks are shown in the Table 1. It can be observed that the mass change of the cured block is closely related to the thermal analysis curve shown in Figure 4. The compressive strength of Mo-PF and the adhesive blocks without heat treatment were 158.16 MPa and 171.28 MPa, respectively. The compressive strength of Mo-PF and the adhesive blocks decreased to 60.98 MPa and 74.59 MPa, respectively, after 400 °C heat treatment in air. When the temperature rose to 600 °C, the overall structure of Mo-PF block was obviously destroyed, and the compressive strength was only 10.42 MPa, while the compressive strength of the adhesive block dropped to 59.32 MPa. Following treatment at 1200 °C, the compression strength of the adhesive block rebounded to 102.41 MPa. In a nitrogen atmosphere, after heat treatment at 400 °C, the compression strength for Mo-PF and the adhesive blocks was 154.04 MPa and 166.01 MPa, respectively. With increasing temperature, these values decreased to 151.05 MPa and 164.62 MPa at 600 °C. After treatment at 1200 °C, the compressive strength reached 34.33 MPa and 56.50 MPa, respectively. With the increase in temperature, the pyrolysis degree of resin increased, and the presence of nitrogen had a certain protective effect on this process. At higher temperatures, the mechanical strength of the adhesive was more dependent on the oxidation of ceramizable fillers.

### 3.5. Compositional Evolution

**Figure 9:**
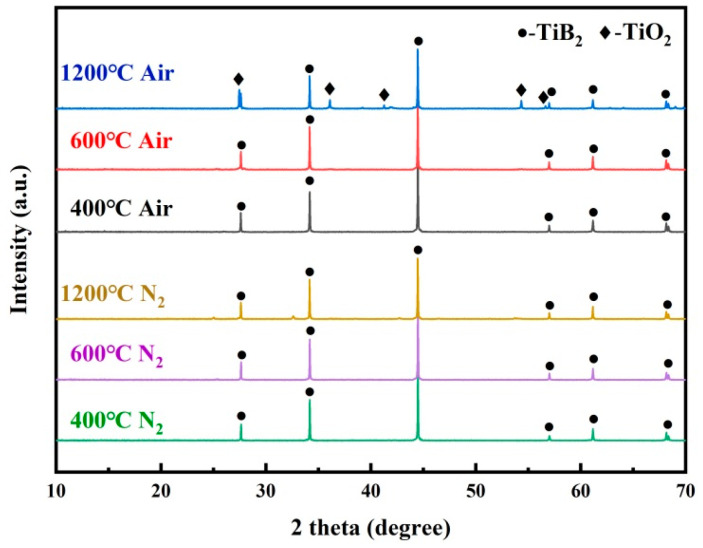
XRD spectra of the adhesive after heat treatment at different temperatures and atmospheres.

In order to better understand the structural evolution of the adhesive with temperature, X-ray diffraction analysis was performed on the adhesive treated at three temperature points, namely 400 °C, 600 °C, and 1200 °C, under different atmospheres. As shown in Figure 9, after heat treatment in air at 400 °C and 600 °C, the main crystalline phase of the adhesive was TiB_2_, which played a crucial role in bearing loads and suppressing crack generation. This positively contributed to the improvement of the joint strength. However, the adhesive bonding strength before this temperature primarily depended on the thermal performance of the resin matrix. When the temperature reached 1200 °C, diffraction peaks of TiO_2_ began to appear, indicating that TiB_2_ acted as active filler, gradually reacting with decomposition products and the atmospheric components to generate TiO_2_ and amorphous B_2_O_3_, filling the defects produced during the resin pyrolysis. This corresponds to the enhancement of the adhesive’s mechanical properties observed in mechanical tests. Adhesive treated in nitrogen only exhibited characteristic peaks of TiB_2_ without the appearance of new crystalline phases. This indicated that TiB_2_ always acts as inert filler in nitrogen with the increase in temperature, thus enhancing the adhesive at the physical level.

## 4. Conclusions

Utilizing molybdenum-modified phenolic resin as the matrix and incorporating TiB2 as the ceramizable filler, a heat-resistant phenolic resin adhesive was prepared and successfully applied in the bonding of Al_2_O_3_ ceramics. The focus was on investigating the changes in the bonding properties and bonding mechanism after different temperature treatments in both air and nitrogen atmospheres. The conclusions are as follows:

1. Before 600 °C, the thermal stability of the adhesive after heat treatment in air and nitrogen was closely related to the pyrolysis behavior of Mo-PF resin. The weight gradually decreased with increasing temperature, but the weight loss rate of the adhesive in nitrogen was lower compared to that in air. After 600 °C, the weight of the adhesive in air started to rise, while the weight change in nitrogen was not significant. In the end, the residual rates of the adhesive at 1200 °C in air and nitrogen were 114.5% and 90.1%, respectively.
2. The bonding strength of Al_2_O_3_ joints after air heat treatment showed a trend of first decreasing (RT–600 °C) and then increasing (600–1200 °C) with increasing treatment temperature, while the bonding strength after nitrogen heat treatment exhibited a slow decrease with the treatment temperature rise. After heat treatment in air and nitrogen at 1200 °C, the shear strength of the Al_2_O_3_ joints was 25.68 MPa and 12.80 MPa, respectively.
3. In air, the pyrolysis of the adhesive matrix resulted in numerous cracks and holes, which were eventually compensated by the ceramic phase formed by the oxidation of TiB_2_ at a high temperature, improving the mechanical properties of the adhesive. In nitrogen, the pyrolysis of the adhesive was slower, and TiB_2_ consistently acted as inert filler, with no apparent oxidation occurring.

## Figures and Tables

**Table 1:** The mass variation and the compressive strength of Mo-PF and the adhesive.

Sample	Mass (g)	Atmosphere	Temperature (°C)	Mass (Heat-Treated) (g)	Compressive Strength (MPa)
Mo-PF	8.53	\	\	\	158.16
	8.14	Air	400	6.91	60.98
	8.71		600	2.47	10.42
	8.12		1200	0.70	0
	8.77	N_2_	400	8.40	154.04
	8.04		600	7.01	151.05
	7.81		1200	5.11	34.33
Adhesive	12.24	\	\	\	171.28
	11.12	Air	400	10.34	74.59
	12.32		600	8.54	59.32
	11.23		1200	12.24	102.41
	13.19	N_2_	400	13.03	166.01
	11.76		600	11.66	164.62
	11.80		1200	9.97	56.50

## Data Availability

Data are contained within the article.
